# Unsupervised Learning in RSS-Based DFLT Using an EM Algorithm

**DOI:** 10.3390/s21165549

**Published:** 2021-08-18

**Authors:** Ossi Kaltiokallio, Roland Hostettler, Hüseyin Yiğitler, Mikko Valkama

**Affiliations:** 1 Unit of Electrical Engineering, Tampere University, 33720 Tampere, Finland; mikko.valkama@tuni.fi; 2 Department of Electrical Engineering, Uppsala University, 75237 Uppsala, Sweden; roland.hostettler@angstrom.uu.se; 3 Department of Communications and Networking, Aalto University, 02150 Espoo, Finland; huseyin.yigitler@aalto.fi

**Keywords:** received signal strength, localization and tracking, bayesian filtering and smoothing, parameter estimation, expectation-maximization algorithm

## Abstract

Received signal strength (RSS) changes of static wireless nodes can be used for device-free localization and tracking (DFLT). Most RSS-based DFLT systems require access to calibration data, either RSS measurements from a time period when the area was not occupied by people, or measurements while a person stands in known locations. Such calibration periods can be very expensive in terms of time and effort, making system deployment and maintenance challenging. This paper develops an Expectation-Maximization (EM) algorithm based on Gaussian smoothing for estimating the unknown RSS model parameters, liberating the system from supervised training and calibration periods. To fully use the EM algorithm’s potential, a novel localization-and-tracking system is presented to estimate a target’s arbitrary trajectory. To demonstrate the effectiveness of the proposed approach, it is shown that: (i) the system requires no calibration period; (ii) the EM algorithm improves the accuracy of existing DFLT methods; (iii) it is computationally very efficient; and (iv) the system outperforms a state-of-the-art adaptive DFLT system in terms of tracking accuracy.

## 1. Introduction

In developed nations, the demographic change in the population is creating many challenges both from a societal and an economic standpoint. Research into aging, age-related conditions, and the means to support an aging population has therefore become a priority for many governments around the world [1]. Ambient assisted living (AAL) is the European Union’s funding program that aims at developing “information and communication technologies (ICT) in a person’s daily living and working environment to enable them to stay active for longer duration, remain socially connected and live independently into old age” (www.aal-europe.eu, accessed on 17 August 2021). This can be achieved by developing both preventive and monitoring systems for aging safely at home, in the community, and at work. In this regard, radio frequency (RF) sensing is particularly suitable for realizing AAL systems. The main advantages of the technology are four-fold:

1. it is passive and does not require users to carry obtrusive and uncomfortable sensors [2];
2. it is suitable for a wide range of monitoring purposes such as localization and tracking [3], fall detection [4], vital sign monitoring [5], gesture recognition [6] and behavioral sensing [7];
3. RF signals can penetrate walls, clutter and other occlusions, unlike many other sensors that have a limited field of view [8]; and
4. it is privacy preserving which increases acceptance of the monitoring technology, unlike vision-based systems that are intrusive [9].

The focus of this paper is on device-free localization and tracking (DFLT), because position and motion embed a wealth of information about people and can be used to develop various AAL applications.

**Figure 1:**
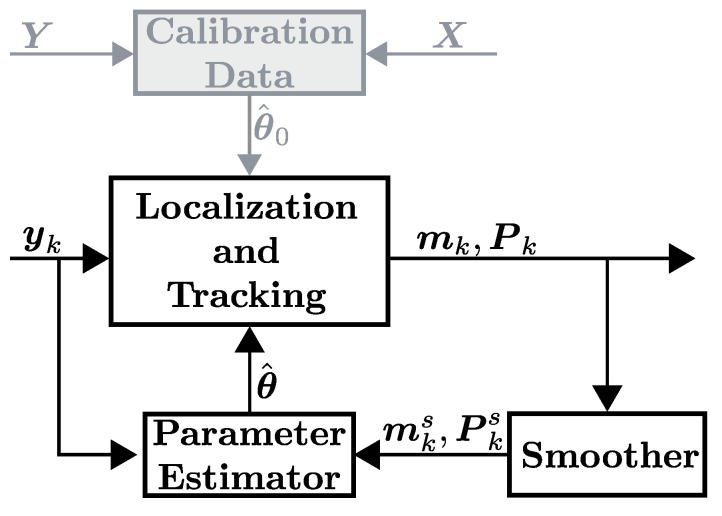
The components of a typical and proposed DFLT system. The gray shaded block and localization-and-tracking block is common to conventional systems, whereas the proposed system is composed of the non-shaded blocks only. The notation is introduced in Section 3 and Section 4.

DFLT methods use received signal strength (RSS) measurements between static wireless nodes to provide location estimates of a person inside the monitored area. In DFLT, there are two fundamental challenges: first, having a model of the RSS as a function of person’s location and, second, maintaining an accurate model over time. Fingerprint-based and data-driven DFLT methods use a supervised training period to collect RSS measurements that are labeled with a person’s known locations [10,11]. The labeled data are used for characterizing the unique propagation properties of the environment. During run time, the non-parametric models can be used to accurately localize people even in challenging indoor deployments. As a drawback, the training process is laborious, and the performance degrades drastically as the environment is altered [12]. Parametric model-based DFLT approaches use physical models to describe the changes in RSS with respect to the locations of the sensors and person [2,13]. Typically, these methods only require a short empty-room calibration period when the area is not occupied by people and hence, they are easier to deploy than fingerprint systems. A downside is that the model inaccuracies limit the localization accuracy and these systems require recalibration and retraining if the environment changes. Both systems can be realized with the Calibration Data unit and Localization-and-Tracking unit shown in Figure 1, and the main difference is how the system is calibrated during the calibration period k∈[a,…,b]. Model-based systems only require the RSS ($Y=[y_{a},\ldots ,y_{b}]$), whereas fingerprinting methods also require the person’s location ($X=[x_{a},\ldots ,x_{b}]$). Considering an AAL application, all empty-room calibration periods and training procedures that require human effort are very inconvenient since it can take from several minutes [2,14] up to half an hour [11,15]. This paper presents a parametric model-based DFLT system that requires no Calibration Data unit, streamlining the requirements for deployment. The proposed system is presented in Figure 1 and the system can be realized using the Localization-and-Tracking unit, the Smoother unit and the Parameter Estimator unit.

**Figure 2:**
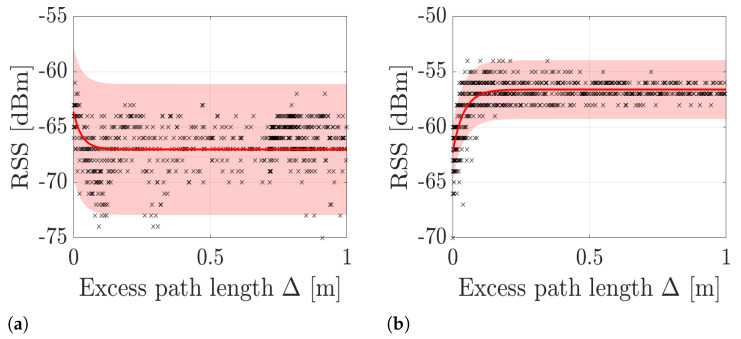
Measured (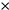) and modeled (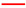) RSS as a function of excess path length for two example links. The shaded region depicts the 3σ confidence interval. In (**a**), the model parameters are Θ=[−67.02,3.42,0.03,3.92] and in (**b**), Θ=[−56.61,−5.71,0.04,0.80].

The work in this paper addresses three major problems associated with the calibration of typical DFLT systems. First, most DFLT methods require an empty-room calibration period when the area is not occupied by people to calculate the mean RSS level μ [2,16]. Typically, the RSS changes are defined with respect to μ and an accurate estimate is a strict requirement of DFLT. Second, it is a common presumption that the model parameters are the same for all links [17,18], i.e., for all transmitter, receiver and frequency channel combinations. Third, model-based DFLT methods that take into account the unique model parameters for each link, require a calibration period when a person moves along a known training trajectory to estimate them [13,19]. If μ is constant and the model parameters are the same for all the links, only a short empty-room calibration period is enough. However, neither of these are valid assumptions in general scenarios. In Figure 2, the measured and modeled RSS as a function of excess path length (see Equation (6)) for two example links is illustrated. As shown, the model parameters of the two links differ significantly from one another invalidating the common assumption. The used model is the exponential model [20] (see Equation (26)) with parameters, $\Theta =[\mu ,\phi ,\lambda ,\sigma^{2}]$, which are estimated using nonlinear least squares over the measurements and known trajectory.

This paper aims to address the limitations and drawbacks of typical DFLT methods by developing a system that does not require separate empty-room calibration periods and that can learn the unique model parameters for each link during the time of operation. The development efforts of this paper are validated using a $75\;m^{2}$ open indoor deployment and a $82\;m^{2}$ residential apartment deployment, and it is shown that the proposed system can achieve an average tracking accuracy as low as 17cm in the open environment and 37cm in the apartment. This paper makes the following contributions:

- A Gaussian filter is presented to estimate the state of the target and a novel Measurement Selection unit is developed to select and combine the measurement models of two DFLT methods into one filtering algorithm. The developed system is demonstrated to outperform a state-of-the-art adaptive DFLT system and reduce the tracking error by 42%.
- A Gaussian smoother is implemented, and it is used to evaluate the expectations involved in the expectation step of the Expectation-Maximization (EM) algorithm. Moreover, we show how the maximization step of the EM algorithm is available in closed form for the considered measurement model. The presented EM algorithm is computationally very efficient, up to 18 times faster than current solutions used in the literature.
- An EM algorithm is presented for estimating the unknown RSS model parameters, liberating the system from the need for supervised training and calibration periods. It is demonstrated that the EM algorithm not only improves the accuracy of the introduced system, but also other DFLT systems.
- The experiments conducted in this paper, together with Matlab code to run the presented filtering, smoothing and EM algorithms are made publicly available and are published in [21]. The aim is to lower the threshold to start research in the area and advance the field of DFLT in general.

The rest of the paper is organized as follows. Related work is discussed in Section 2. Section 3 formulates the problem and presents the localization-and-tracking system. The parameter estimation framework is presented in Section 4. The experiments that were conducted are introduced in Section 5 and the results are presented in Section 6. Thereafter, conclusions are drawn.

## 2. Related Work

In this section, related works that use calibration and training are summarized. We begin the section by introducing the most common method in DFLT—using an empty-room calibration period. Systems that use online training are discussed thereafter. Lastly, works that calibrate the model using supervised or unsupervised training are presented. Empty-room calibration—Most DFLT systems define the RSS changes with respect to μ, often referred to as the reference or baseline RSS. The system performance depends on an accurate estimate of μ and therefore, it is typically calculated over several minutes when the area is not occupied by people [2,18,22]. In this paper, μ is one of the parameters estimated by the EM algorithm and it is shown that the system can be initialized without having an empty-room calibration period.

It is worth noting that methods that do not define the RSS changes with respect to μ have also been proposed [8,23]. Instead, these methods calculate the sample RSS variance over a fixed time window and do not require calibration. A downside is that variance-based methods cannot locate stationary targets because in such scenarios, the variance drops close to the noise floor and does not show up in the estimated image.

Online calibration—Several works have proposed calibrating the reference RSS μ [14,24], or measurement noise variance $\sigma^{2}$ [25,26] online. The system can be deployed without a calibration period when the reference RSS is estimated on-the-fly. Moreover, improved estimators can be developed when better noise models are available [23,25,26]. These approaches first estimate the target state and then the parameters in a sequential order. However, such a decoupling is not always possible, or degrades the system performance if the state estimates are inaccurate. Unlike online calibration methods, this work uses the EM algorithm to estimate μ and $\sigma^{2}$ from batches of data that is collected while the person is inside the monitored area.

Estimating the RSS distribution (defined by μ and $\sigma^{2}$) in two states, when a person is crossing or not crossing the imaginary link line between the transceivers, has been explored in [27,28,29,30,31]. Not only can this information be used to localize people, but also to determine if the monitored area is occupied—a non-trivial task in RF sensing. The aforementioned works model the target location as a binary state: either the person is in between the transmitter (TX) and receiver (RX), or they are not. In this paper, we use a continuous measurement model and there is no need to explicitly determine whether a target is crossing the link or not.

Model calibration—The works in [13,15,19] use an offline calibration phase for estimating parameters of the measurement model. During calibration of the parameters, a person moves along a known training trajectory and visits locations of interest. The RSS is recorded between the static wireless nodes and the measurement model parameters are estimated. During the online phase, the calibrated measurement model is used in the tracking algorithm. The works cited above demonstrate high tracking accuracy, but the calibration phase is inconvenient since it can take up to 30 min as in [15]. In this paper, the estimated trajectory and EM algorithm are used for unsupervised learning. The position estimates can be inaccurate in the beginning, but as the person moves in the area, the model parameters can be estimated more accurately resulting in improved tracking performance. The proposed parameter estimation method does not require human intervention other than normal movement in the area.

Parameter estimation—This present work is most closely related to the developments in [22,32], which have an online calibration module as well as a batch-estimation module for tuning the model parameters. The aforementioned works use an imaging solution, and the accuracy is inevitably affected by the binary measurement model and resolution of the pixels. Instead, we use a continuous measurement model, and the implemented Bayesian filter directly estimates the kinematic state of the target using the RSS and higher tracking accuracy is expected [20]. Furthermore, the existing approaches use a nonlinear least-squares solution to estimate the model parameters [22,32], while the proposed method solves the problem using a maximum likelihood approach based on a computationally efficient EM algorithm. Since unsupervised learning depends on accurate position estimates, the introduced system is superior with respect to [22,32]. The experimental results demonstrate that the proposed system can reduce the tracking error by 42% or more and the EM algorithm is computationally up to 18 times faster than the nonlinear optimization method used in [22].

Expectation-maximization has also been used for RSS-based DFLT in [20,33]. However, these approaches use a mini-batch- and particle-filtering-based online EM approach. Although the online EM approach is attractive for on-the-fly estimation of the parameters and rapid adaptation to changing environments, the method also suffers from some important drawbacks. First, since the expectation step is based on particle filtering, which yields a degenerate approximation of the smoothing posterior density required by EM [34,35], it is computationally heavy, and the estimation of the marginal log-likelihood may be poor. Second, in [20,33] the maximization step cannot be done in closed form, and it is implemented by propagating a set of sufficient statistics and the numerical integration is carried out using importance sampling. In contrast, in this paper, the expectation step is calculated using a Gaussian approximation for the smoothing distribution which can be computed efficiently and does not suffer from trajectory degeneration. Furthermore, we show that the maximization step of the EM algorithm is available in closed form for the considered measurement model and implemented Gaussian smoother. Hence, with respect to [20,33], the solution presented in this paper is more tractable in terms of the approximation of the expectation and maximization steps and computational complexity. In addition, the EM algorithm used in [20,33] is only evaluated with simulations, whereas we validate our proposed method using experimental data. The EM algorithm is widely used in different applications and the readers are referred to [36] for an introduction to parameter estimation and to [37,38] for a more general treatment of parameter estimation in nonlinear dynamical system using Gaussian filtering and smoothing.

## 3. Localization and Tracking

**Figure 3:**
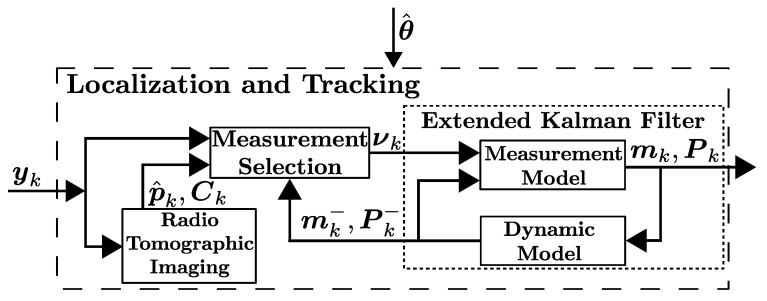
Major components of the proposed localization-and-tracking method. The notation is introduced in Section 3 and Section 4.

This work aims to track the kinematic state of a target using the RSS measured between static wireless nodes. The components of the introduced Localization-and-Tracking unit and their relations are visualized in Figure 3 and presented in the following. We begin by presenting the models used for localization and tracking. Thereafter, the estimation tasks are presented, and they are performed by two complementary blocks: (i) the Radio Tomographic Imaging (RTI) unit summarized in Section 3.2, and (ii) the Extended Kalman Filter (EKF) unit presented in Section 3.3. The EKF uses the combination of RSS and RTI position estimates in the measurement update, and the Measurement Selection unit selects and combines the measurements as described in Section 3.4.

The idea to augment the EKF with RTI position estimates was originally presented in [3]. The localization-and-tracking system presented in this paper further improves the filter by introducing a Measurement Selection unit which selects and combines the measurements in a way that enhances the tracking accuracy. In addition, we propose a novel RTI positioning scheme that also estimates covariance of the position estimates. Furthermore, the developed localization-and-tracking system performs no low-pass filtering of the RTI images in contrast with the system presented in [3]. The reason is that image filtering has a negative impact on the parameter estimation algorithm since it introduces a lag in the state estimates and causes correlated position errors.

### 3.1. Models

#### 3.1.1. Dynamic Model

For DFLT, the state of the system in two-dimensional Euclidean space can be defined as

(1) $$x_{k}={\left[\begin{array}{cccc} x_{k} & {\dot{x}}_{k} & y_{k} & {\dot{y}}_{k} \end{array}\right]}^{T}$$

where $x_{k}$ and $y_{k}$ are the *x*- and *y*-coordinates, and the velocity components are denoted as ${\dot{x}}_{k}$ and ${\dot{y}}_{k}$. This state representation is particularly suitable for DFLT because the position and velocity define the temporal and spectral properties of the RSS [39]. This state evolves at time *k* in accordance with

(2) $$x_{k}=Fx_{k-1}+q_{k-1},$$

where F is the state transition matrix of the dynamic model and $q_{k-1}∼N\left(0,Q\right)$ is Gaussian process noise. As a person is not expected to change velocity very rapidly and unexpectedly, a common choice of F in RSS-based DFLT [18,22] is the second-order kinematic model [40], given by

(3) $$F=I_{2}⊗\left[\begin{array}{cc} 1 & \tau \\ 0 & 1 \end{array}\right],\;Q=I_{2}⊗q\left[\begin{array}{cc} \frac{1}{3}\tau^{3} & \frac{1}{2}\tau^{2}\\ \frac{1}{2}\tau^{2} & \tau \end{array}\right]$$

where $I_{2}$ is an identity matrix, ⊗ the Kronecker product, *q* the power spectral density of the process noise and τ the sampling period.

#### 3.1.2. Measurement Model

Consider a wireless network, where each of the *S* nodes can communicate with the other S−1 nodes. Moreover, the wireless devices can communicate on *C* different frequency channels. Each transmitter, receiver and channel combination is a unique link and the total number of measured links is L=C·S·(S−1). It is to be noted that full connectivity is not mandatory for DFLT. It is also to be noted that we do not assume channel reciprocity. The reason being, although the radio channel is reciprocal, measurements of the radio channel are not reciprocal, and parameters of the reciprocal link can be different [41].

The nodes communicate in round-robin fashion and at time *k*, one node transmits and the other nodes receive. At the next time instant, k+1, the transmission turn is assigned to the next node in the schedule. The nodes transmit sequentially, and one communication cycle consists of one transmission by every node. At the end of the communication cycle, the nodes switch simultaneously to the next frequency channel in a predefined list. Thereafter, a new communication cycle is initiated. Once each node has transmitted on every frequency channel, the schedule is restarted from the beginning.

For the considered problem, the measurement system at time *k* can be defined as

(4) $$y_{k}=I_{k}\left(H\left(x_{k}\right)\theta +r_{k}\right),$$

where $y_{k}\in R^{(S-1)\times 1}$ is the measured RSS, $I_{k}\in R^{(S-1)\times L}$ a deterministic link indicator matrix defined by the schedule (see Section 3.3.1), $H(x_{k})\theta$ the linear-in-parameters measurement model and $r_{k}∼N\left(0,R\right)$ is Gaussian measurement noise. The human-induced RSS changes are modeled using an exponential model [20] and the complete linear-in-parameters model is defined as

(5) $$\begin{array}{cc} H(x_{k}) & =\left[\begin{array}{ccccccc} 1 & \;e^{-\Delta_{1,k}/\lambda } & 0 & \;0 & \; & & \\ 0 & \;0 & 1 & \;e^{-\Delta_{2,k}/\lambda } & \; & & \\ \; & \; & & \; & \ddots & \; & \\ \; & \; & & \; & & 1 & \;e^{-\Delta_{L,k}/\lambda } \end{array}\right]\;,\\ \theta & ={\left[\begin{array}{ccccccc} \mu_{1} & \phi_{1} & \mu_{2} & \phi_{2} & \ldots & \mu_{L} & \phi_{L} \end{array}\right]}^{T}\;, \end{array}$$

where λ is the decay rate, $\mu_{l}$ the reference RSS and $\phi_{l}$ the measurement gain, $H\left(x_{k}\right)\in R^{L\times 2L}$ and $\theta \in R^{2L\times 1}$. In (5), the excess path length $\Delta_{l,k}$ defines an ellipse with the foci at the TX and RX and it relates the person’s location $p_{k}={\left[\begin{array}{cc} x_{k} & y_{k} \end{array}\right]}^{T}$ to link *l* with TX *m* and RX *n* by

(6) $$\Delta_{l,k}≜∥p_{m}-p_{k}∥+∥p_{n}-p_{k}∥-∥p_{m}-p_{n}∥,$$

where $p_{m}$ and $p_{n}$ denote the TX and RX positions in respective order. Lastly, the measurement noise covariance is assumed diagonal and it is defined as $R=\mathrm{diag}\left(\sigma_{1}^{2},\;\sigma_{2}^{2},\;\ldots ,\sigma_{L}^{2}\right)$. It is to be noted that the RSS can be measured at most for S−1 links simultaneously because only one node transmits at a time. Moreover, to measure the *L* links takes S·C transmissions and S·C·τ duration of time.

### 3.2. Radio Tomographic Imaging

#### 3.2.1. Image Estimation

RTI estimates a discretized RSS change field, denoted by $b_{c}$, using the RSS of J=S(S−1) links measured on frequency channel *c*. As in [42], the RSS is assumed to be a linear combination of voxel changes plus noise

(7) $$z_{c}=W_{c}b_{c}+r_{c},$$

where $z_{c}\in R^{J\times 1}$ is the mean-removed RSS, $W_{c}\in R^{J\times N}$ a weight matrix that relates the spatial change field $b_{c}\in R^{N\times 1}$ to the RSS, *N* the voxel number and $r_{c}\in R^{J\times 1}$ the measurement noise. The measurement vector and noise covariance in (4) can be decomposed as $y={\left[\begin{array}{ccc} y_{1}^{T} & y_{2}^{T} & \ldots y_{C}^{T} \end{array}\right]}^{T}$ and $R=\mathrm{diag}\left(R_{1},\;R_{2},\;\ldots ,R_{C}\right)$ where $y_{c}$ and $R_{c}$ denote the RSS and measurement noise covariance on channel *c*. Now, the RTI measurement and noise vectors are related to the model in (4) via $z_{c}=y_{c}-\mu_{c}$ and $r_{c}∼N\left(0,R_{c}\right)$.

The minimum mean square error estimate for the model in (7), with zero-mean Gaussian image prior $b∼N(0,\Sigma_{b})$, is

(8) $$\begin{array}{cc} {\hat{b}}_{c} & =\Pi_{c}z_{c},\;\mathrm{where}\\ \Pi_{c} & ={\left(W_{c}^{T}R_{c}^{-1}W_{c}+\Sigma_{b}^{-1}\right)}^{-1}W_{c}^{T}R_{c}^{-1}. \end{array}$$

The covariance matrix $\Sigma_{b}$ for pixels *m* and *n* is [42]

(9) $${\left\{\Sigma_{b}\right\}}_{m,n}=\sigma_{b}^{2}\exp\left(\frac{-∥p_{m}-p_{n}∥}{\delta_{d}}\right),$$

where $\sigma_{b}^{2}$ is the variance of each pixel and $\delta_{d}$ is a user-defined space constant. For link *l* and pixel *n*, we define the elements of $W_{c}$ as

(10) $${\left\{W_{c}\right\}}_{l,n}=\frac{\phi_{l}}{∥\phi_{l}∥}e^{-\Delta_{l,n}/\lambda },$$

where $\phi_{l}$ and λ are the measurement gain and decay rate of the model defined in (5), $∥\phi_{l}∥$ is a normalization term and $\Delta_{l,n}$ the excess path length. In the literature, W has taken many forms, and the reader is referred to [16,43] for further details.

The projection matrix $\Pi_{c}$ is channel dependent and it is computed independently for each of the channels. However, $\Pi_{c}$ must be computed only once at the beginning of the experiment and the real-time computation of the image requires only one matrix multiplication, of O[NL] multiplications and additions. The spatial change field is estimated at the end of each communication cycle when kmodS=0 and $z_{c}$ contains measurements from time instant k−S+1 to *k*. The image estimate on channel *c* is denoted from now on as ${\hat{b}}_{k}$, to prevent using two time notations.

#### 3.2.2. RTI Positioning

For a single target, localizing the person can be postulated as finding the mode of ${\hat{b}}_{k}$ since it is expected that the pixels with highest intensity locate near the target [44]. The mode is in the set of pixels with intensity higher than γB, where $B=\max({\hat{b}}_{k})$ denotes the maximum component of ${\hat{b}}_{k}$ and 0≤γ≤1 is a threshold. The threshold is a tuning parameter between two extremes: if γ=0 all pixels are taken into account and if γ=1 only a single pixel is accounted for, and we have empirically found that γ=0.7 provides a good overall performance. Let us define

(11) $${\tilde{b}}_{k}=\left\{\begin{array}{cc} {\hat{b}}_{k} & \mathrm{if}\;{\hat{b}}_{k}\geq \gamma B\\ 0 & \mathrm{otherwise} \end{array}\right.,$$

where $w={\tilde{b}}_{k}/\sum {\tilde{b}}_{k}$. Now, the position estimate and sample covariance are given by:

(12) $$\begin{array}{cc} {\hat{p}}_{k} & =\sum_{n=1}^{N}w_{n}p_{n}, \end{array}$$

(13) $$\begin{array}{cc} C_{k} & =\sum_{n=1}^{N}w_{n}\left(p_{n}-{\hat{p}}_{k}\right){\left(p_{n}-{\hat{p}}_{k}\right)}^{T}, \end{array}$$

where $p_{n}={\left[\begin{array}{cc} x_{n} & y_{n} \end{array}\right]}^{T}$ are the pixel coordinates and $w_{n}$ the weight for pixel *n*.

**Figure 4:**
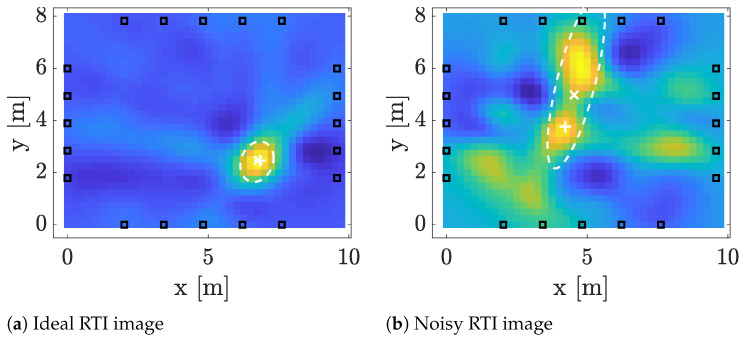
Two example RTI images and the position and covariance estimates calculated using (12) and (13). In the image, the deep blue regions indicate areas that are not occupied by people and the bright regions indicate estimated obstructions. Furthermore, the plus sign indicates the true position, the crosses are the position estimates, and the dashed line illustrates the 3σ uncertainty ellipse [3], reprinted with permission from ref. [3]. Copyright 2019 IEEE.

An example is illustrated in Figure 4 in which two RTI images are shown together with the estimates given in Equations (12) and (13). The image on the left is an ideal RTI image, pixels with ${\hat{b}}_{k}\geq \gamma B$ are centered around the target location and the image has very little noise resulting in an accurate position estimate and small covariance. The image on the right is very noisy and the image is multimodal. As a result, the estimated position does not accurately indicate the target location and the estimated covariance is significantly higher than in the other image. However, estimating the covariance allows taking such uncertainties into account and the tracking filter developed in the next section gives less weight to position estimates that are estimated from noisy images, such as the one on the right of Figure 4.

### 3.3. Tracking Filter

The extended Kalman filter (EKF) computes the marginal posterior distribution of $x_{k}$ for each time step *k* using the data $y_{1},\ldots ,y_{k}$ and assuming Gaussian approximations for the filtering densities so that $p\left(x_{k}|y_{1:k}\right)\approx N\left(x_{k}|m_{k},P_{k}\right)$. Different than conventional Bayesian filtering implementations for DFLT [13,18,20], in this work, the measurement model of the filter is augmented with the position estimates from RTI as in [3]. This bounds the filter’s measurement residuals by the position errors of the imaging approach. Therefore, the developed filter has the robustness of an imaging method and the tracking accuracy of a Bayesian filter. The filtering algorithm consist of three steps: (i) prediction step, (ii) model selection, and (iii) measurement update step. We simply refer to the introduced filter as EKF, although it is more complex than a first-order filter that would solely use RSS. In the following, we first present the observation model of the EKF and thereafter, the prediction and update steps of the filter.

#### 3.3.1. EKF Observation Model

Recall that at a given time instant *k*, at most S−1 links are measured. Instead of using the complete model defined in Equation (5), the EKF operates on a subset of the measurement model. We refer to the subset as the *observation model* and essentially, it contains the measurements and associated models sampled at time *k*. Thus, the observation model is defined by the TX and channel identifiers, and it changes with time. To explicitly define the observation model, consider the set of nodes S={1,2,…,S} and the set of channels C={1,2,…,C}. Then, the link index *l* corresponding to the transmission by node i∈S on frequency channel c∈C and received by node j∈S is

$$l=\left\{\begin{array}{cc} (c-1)S(S-1)+(i-1)S+j-i & i<j,\\ (c-1)S(S-1)+(i-1)S+j-i+1 & i>j. \end{array}\right.$$

The RXs that measure the RSS at time k are R=S∖{i} and |R|=S−1. Now if m=[1,…,|R|], we can define the indices of the link selection matrix, measurement model, and noise covariance as follows:

(14) $$\begin{array}{cc} {\left\{I_{k}\right\}}_{m,l} & =1,\\ {\left\{H\left(x_{k}\right)\theta \right\}}_{m,1} & ={\left\{H\left(x_{k}\right)\theta \right\}}_{l,1},\\ {\left\{R\right\}}_{m,m} & ={\left\{R\right\}}_{l,l}. \end{array}$$

In addition, the EKF requires the Jacobian of $H(x_{k})\theta$, and the elements of this matrix are given by

(15) $${\left[\begin{array}{cc} \frac{\partial {\left\{H\left(x_{k}\right)\theta \right\}}_{m,1}}{\partial x} & \frac{\partial {\left\{H\left(x_{k}\right)\theta \right\}}_{m,1}}{\partial y} \end{array}\right]}^{T}=\frac{{\left\{H\left(x_{k}\right)\theta \right\}}_{m,1}}{\lambda }\left(\frac{p_{i}-p_{k}}{∥p_{i}-p_{k}∥}+\frac{p_{j}-p_{k}}{∥p_{j}-p_{k}∥}\right),$$

where $p_{i}$ and $p_{j}$ denote the positions of nodes *i* and *j*. The Jacobian for *m* is

(16) $${\left\{H_{x}\right\}}_{m,\cdot }=\left[\begin{array}{cccc} \frac{\partial {\left\{H\left(x_{k}\right)\theta \right\}}_{m,1}}{\partial x} & 0 & \frac{\partial {\left\{H\left(x_{k}\right)\theta \right\}}_{m,1}}{\partial y} & 0 \end{array}\right].$$

#### 3.3.2. Prediction Step

Given that the dynamic model in (2) is linear, the prediction step of the first-order additive noise EKF can be expressed as [36]

(17) $$\begin{array}{cc} m_{k}^{-} & =Fm_{k-1},\\ P_{k}^{-} & =FP_{k-1}F^{T}+Q, \end{array}$$

where $m_{k}$ and $P_{k}$ denote the state estimate and covariance in respective order, and $m_{k}^{-}$ and $P_{k}^{-}$ are the predicted mean and covariance.

#### 3.3.3. Measurement Update

The *measurement selection* unit presented in Section 3.4 calculates the measurement residual $\nu_{k}$ and forms the associated measurement noise covariance matrix R and measurement model matrix H. Using these, the mean $m_{k}^{-}$ and covariance $P_{k}^{-}$ can be updated using [36]

(18) $$\begin{array}{cc} S_{k} & =HP_{k}^{-}H^{T}+R,\\ K_{k} & =P_{k}^{-}HS_{k}^{-1},\\ m_{k} & =m_{k}^{-}+K_{k}\nu_{k},\\ P_{k} & =P_{k}^{-}-K_{k}S_{k}K_{k}^{T}. \end{array}$$

### 3.4. Measurement Selection

The DFLT system implementations using Bayesian filtering or imaging (in particular RTI) have different characteristics. Depending on the target’s position and system deployment, the performance of the introduced filter can be improved by enabling or disabling certain measurements. For example, the covariance of the RTI position estimate can be small and biased. On the other hand, the filter can converge to an incorrect trajectory and the estimated covariance is not able to account for the uncertainties in the state estimate. To solve these issues, a logic to select the effective measurements is introduced. The procedure is based on the normalized innovation squared (a.k.a. square of the Mahalanobis distance) [40]

(19) $$\epsilon_{1}={\left({\hat{p}}_{k}-Hm_{k}\right)}^{T}{\left(HP_{k}H^{T}+C_{k}\right)}^{-1}\left({\hat{p}}_{k}-Hm_{k}\right),$$

where

(20) $$H=\left[\begin{array}{cccc} 1 & 0 & 0 & 0\\ 0 & 0 & 1 & 0 \end{array}\right]$$

is the linear measurement model. The test statistic has a $\chi^{2}$ distribution with two degrees of freedom and it can be used to assess whether the realized RTI estimate is unexpectedly large with respect to the prior predictive distribution. In addition, the square of the Mahalanobis distance between two successive RTI estimates is calculated

(21) $$\epsilon_{2}={\left({\hat{p}}_{k}-{\hat{p}}_{k-S}\right)}^{T}{\left(C_{k}+C_{k-S}\right)}^{-1}\left({\hat{p}}_{k}-{\hat{p}}_{k-S}\right),$$

where ${\hat{p}}_{k-S}$ and $C_{k-S}$ denote the previous RTI position estimate and covariance. The test statistic can be used to assess whether the prior predictive distribution has converged to an incorrect trajectory.

For simplicity, the index notation is dropped and the measurement model, measurement noise covariance matrix, and Jacobian are simply denoted as: $H\left(x_{k}\right)\theta \in R^{|R|\times 1}$, $R\in R^{\left|R|\times |R\right|}$ and $H_{x}\in R^{|R|\times 4}$, respectively. The resulting logic to select the measurement models is presented below in which T denotes the confidence interval of the $\chi^{2}$ distribution with two degrees of freedom.

- **if**$\epsilon_{1}>T$ and $\epsilon_{2}\leq T$—It is likely that the filter has diverged. Use only the output of RTI, i.e., $R=C_{k}$, H=H and $\nu_{k}={\hat{p}}_{k}-Hm_{k}^{-}$.
- **else if**$\epsilon_{1}\leq T$—Normal operation, concatenate the models: $R=\mathrm{blkdiag}\left(C_{k},R\right)$, $H={\left[\begin{array}{cc} H^{T} & H_{x}^{T} \end{array}\right]}^{T}$ and $\nu_{k}={\left[\begin{array}{cc} {\left({\hat{p}}_{k}-Hm_{k}^{-}\right)}^{T} & {\left(y_{k}-H\left(m_{k}^{-}\right)\theta \right)}^{T} \end{array}\right]}^{T}$
- **else**—The RTI position estimate is likely inaccurate, use only the RSS measurements, i.e., R=R, $H=H_{x}$ and $\nu_{k}=y_{k}-H\left(m_{k}^{-}\right)\theta$.

The measurement residual $\nu_{k}$, measurement noise covariance matrix R and measurement model matrix H are used by the EFK update step presented in Section 3.3.3.

## 4. Parameter Estimation

In this section, an EM algorithm based on Gaussian smoothing is developed. The section begins by introducing the Gaussian smoothing recursion for the considered problem. Thereafter, we show how the developed Gaussian smoother can be used to evaluate the expectations involved in the E-step of the EM algorithm. We also derive the solution of the maximization problem in the M-step in closed form for the considered measurement model.

### 4.1. Gaussian Smoothing

In this section, we present the smoothing recursions for the Rauch-Tung-Striebel smoother (RTSS). The RTSS computes the marginal posterior distribution of the state by conditioning on the whole measurement data. The smoothing solution is given by $p\left(x_{k}|y_{1:K}\right)\approx N\left(x_{k}|m_{k}^{s},P_{k}^{s}\right)$, where K≥k, which can be calculated recursively. The smoothing recursion starts from the last time step *K* and proceeds backwards to the first time step, and the recursion is given by [36]

(22) $$\begin{array}{cc} P_{k+1}^{-} & =FP_{k}F^{T}+Q,\\ G_{k} & =P_{k}F^{T}{\left(P_{k+1}^{-}\right)}^{-1},\\ m_{k}^{s} & =m_{k}+G_{k}\left(m_{k+1}^{s}-Fm_{k}\right),\\ P_{k}^{s} & =P_{k}+G_{k}\left(P_{k+1}^{s}-P_{k+1}^{-}\right)G_{k}^{T}. \end{array}$$

In the EM algorithm, the expectation is over the smoothing distribution (see Equation (24)) and the obtained smoother result is used by the EM algorithm.

### 4.2. Expectation-Maximization-Based Parameter Estimation

Likelihood-based parameter estimation approaches seek to estimate the unknown model parameters Θ from the marginal likelihood $p\left(y_{1:K}|\Theta \right)$. In general, these methods maximize the logarithm of $p\left(y_{1:K}|\Theta \right)$ to find the maximum likelihood (ML) estimate of Θ, given by

(23) $$\Theta =\underset{\Theta }{arg max}\left[\log p\left(y_{1:K}|\Theta \right)\right].$$

The likelihood is over the joint density of the measurements and the latent state variables. Since computation of the high-dimensional integral required in marginalizing the states out is practically impossible, in this paper, we use the EM for approximating ML estimation. The key idea behind the EM algorithm is that the marginal likelihood can be maximized by iteratively maximizing its lower bound, which is equivalent to maximizing [36]

(24) $$Q\left(\Theta ,\Theta^{\left(i\right)}\right)=E\left\{\log p\left(x_{0:K},y_{1:K}|\Theta \right)|y_{1:K}\right\},$$

where the expectation is with respect to $p(x_{0:K}|y_{1:K},\Theta^{\left(i\right)})$ and $\Theta^{\left(i\right)}$ denotes the parameter estimate at iteration *i*. The expectation step of the EM algorithm is equivalent to computing (24) over the smoothing distribution, whereas the maximization step aims at maximizing $Q(\Theta ,\Theta^{\left(i\right)})$ with respect to Θ.

Using the properties of the state-space model and noting that the parameters only enter the measurement likelihood, maximizing Q is equivalent to maximizing (see, e.g., [36,38])

(25) $$\tilde{Q}\left(\Theta ,\Theta^{\left(i\right)}\right)=\sum_{k=1}^{K}E\left\{\log p\left(y_{k}|x_{k},\Theta \right)|y_{1:K}\right\}.$$

Furthermore, for the DFLT problem considered in this paper and under the assumption that the measurement noises of the individual links are mutually independent, zero-mean Gaussian noise, $r_{l,k}∼N\left(0,\sigma_{l}^{2}\right)$ and $\mathrm{Cov}\{r_{l},r_{j}\}=0,\;\forall \;l\neq j$, the parameters can be estimated independently for each of the *L* links as follows. First, recall that the measurement model of the *l*th link is given by

(26) $$\begin{array}{cc} y_{l,k} & =\left[\begin{array}{cc} 1 & e^{-\Delta_{l,k}/\lambda } \end{array}\right]\left[\begin{array}{c} \mu_{l}\\ \phi_{l} \end{array}\right]+r_{l,k}\\ & =h_{l}\left(x_{k}\right)\theta_{l}+r_{l,k}. \end{array}$$

Then, it is shown in Appendix A that the M-step maximizing the approximation of $\tilde{Q}\left(\Theta ,\Theta^{\left(i\right)}\right)$ in (25) has a closed form solution and the parameter update is given by

(27) $$\begin{array}{cc} {\hat{\theta }}_{l} & =G^{-1}B,\\ {\hat{\sigma }}_{l}^{2} & =\frac{1}{K}\left(D-B^{T}G^{-1}B\right), \end{array}$$

where *K* is the number of measurements of link *l*, and B, D and G are calculated using the latest smoother results as follows

(28) $$\begin{array}{cc} B & =\sum_{k=1}^{K}E{\left\{h_{l}\left(x_{k}\right)|y_{1:K}\right\}}^{T}y_{l,k},\\ D & =\sum_{k=1}^{K}y_{l,k}^{T}y_{l,k},\\ G & =\sum_{k=1}^{K}E{\left\{h_{l}{\left(x_{k}\right)}^{T}h_{l}\left(x_{k}\right)|y_{1:K}\right\}}^{T}. \end{array}$$

The expectations used to calculate B and G involve nonlinear transformations of $x_{k}$ which can be approximated using Taylor series expansion. This yields

(29) $$\begin{array}{cc} E\left\{h_{l}\left(x_{k}\right)|y_{1:K}\right\} & \;\approx \;h_{l}\left(m_{k}^{s}\right),\\ E\left\{h_{l}{\left(x_{k}\right)}^{T}h_{l}\left(x_{k}\right)|y_{1:K}\right\} & \;\approx \;h_{l}{\left(m_{k}^{s}\right)}^{T}h_{l}\left(m_{k}^{s}\right)\;+\;H_{x}^{T}P_{k}^{s}H_{x}, \end{array}$$

where $m_{k}^{s}$ and $P_{k}^{s}$ are the mean and covariance of the smoother result and $H_{x}$ is the Jacobian of $h_{l}$ evaluated at $m_{k}^{s}$ (see Equation (16)). For a more detailed treatment of EM-based parameter estimation please refer to, for example, [36,37,38].

Due to quantization of the RSS, the estimated variance may be zero even though the true real-valued received power would have had a positive variance. We apply shrinkage, which imposes an L2-penalty on the estimated covariance matrix, to assure positive variance and avoid numerical instability. In practice, the L2-penalized ML estimate is given by the simple convex transformation:

(30) $$R=\left(1-\alpha \right)\hat{R}+\alpha \frac{\mathrm{Tr}\;\hat{R}}{L}I_{L},$$

where $\hat{R}=\mathrm{diag}\left({\hat{\sigma }}_{1}^{2},\;{\hat{\sigma }}_{2}^{2},\;\ldots ,{\hat{\sigma }}_{L}^{2}\right)$, α is the shrinkage coefficient, $\mathrm{Tr}\;\hat{R}$ denotes the trace of the matrix and $I_{L}$ is an identity matrix.

## 5. Experiments

The development efforts of this paper are demonstrated using Texas Instruments CC2531 USB dongle nodes [45]. The nodes operate on the 2.4 GHz ISM band and communicate on a set of frequency channels C∈{11,…,26} defined by the IEEE 802.15.4 standard [46]. The wireless nodes follow a round-robin schedule as discussed in Section 3.1.2. In the transmitted packets, the nodes include the most recent RSS measurements, associated with the transmissions of other nodes. The time interval between the communications is approximately, τ≈2.9ms, defining the sampling period for the system. A base station that overhears all the traffic extracts the RSS from the packets and relays the measurements to a computer through UART for centralized processing. The readers are referred to [47] for a detailed description of the communication protocol. It is to be noted that the method of this paper can be generalized to any device capable of measuring the RSS including Wi-Fi, Bluetooth and RFID.

**Figure 5:**
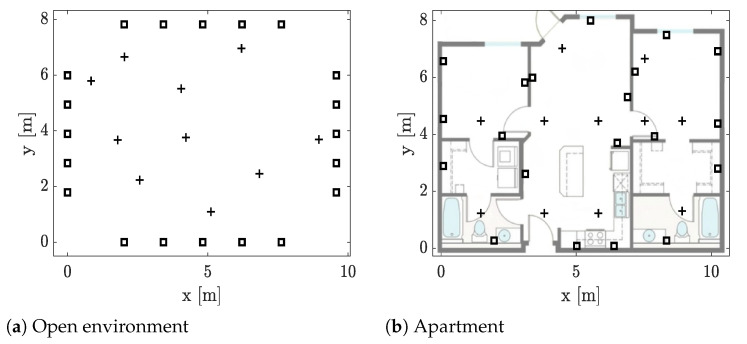
The experimental layouts in which the nodes (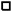) and the reference positions (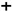) are illustrated [3], reprinted with permission from ref. [3]. Copyright 2019 IEEE.

The experiments are conducted in an open indoor environment and in a downtown residential apartment. In both experiments, 20 nodes are deployed as illustrated in Figure 5. In the open environment, the nodes are set on top of podiums (≈0.9 m) and deployed around a $75\;m^{2}$ area. The size of the apartment is $82\;m^{2}$ and the nodes are deployed by the electric sockets so they could be powered from the mains. The walk-in closets did not have electric sockets on the exterior walls, so we decided to deploy one battery-powered node in each to ensure coverage of the entire apartment. These two nodes are located at ${\left[0.08\;2.89\right]}^{T}$ and ${\left[10.24\;2.80\right]}^{T}$.

Before the experiment, reference positions were defined and marked. During the experiment, the person’s trajectory follows the imaginary lines between the markers. Once the target reaches a reference position, they stop, remain stationary for a few seconds, and then walk to the next reference position. During the experiment, the person is carrying a video camera. In post-processing, the RSS and video streams are synchronized, and the video is used to define the ground truth trajectory. In Section 6.4, the statistical significance of the tracking error is tested to assure that the generated trajectory is close to the ground truth.

The experiments in both environments are conducted with one, four and 16 frequency channels and the set of used channels are: C={26}, C={11,16,21,26} and C={11,…,26}. In addition, three different trials are conducted with each channel number. The trials are approximately three minutes long and every reference position is visited at least once in each trial. In the following section, the experiments are referred to as Exi.j., where *i* indicates the experiment number and *j* the trial. Experiments 1–3 are conducted in the open environment and experiments 4–6 in the apartment. Furthermore, Ex1 and Ex4 use one channel, Ex2 and Ex5 four channels, and Ex3 and Ex6 all 16 frequency channels. In the apartment experiment, there are several co-existing Wi-Fi networks located in the coverage area, but the presented system can remain operational. The system is not particularly sensitive to occasional packet drops and frequency channel diversity partly mitigates interference issues. As an example, the packet reception rate is below 85% on the most congested channel and above 99% on channels that do not share the frequency band with Wi-Fi.

The imaging parameters used in the experiments are given in Table 1, whereas the parameters of the tracking algorithm are defined by the measurement model $\Theta =[\mu ,\phi ,\lambda ,\sigma^{2}]$. In the experiments, λ=0.04m is assumed to be constant unless otherwise stated, the measurement gain and variance are initialized using $\phi_{0}=-5\;\mathrm{dB}$ and $\sigma_{0}^{2}=1\;{\mathrm{dB}}^{2}$. In Section 6, the initialization of $\mu_{0}$ is discussed. The only user-defined parameter in the EKF is the process noise value and it should be tuned to the actual motion. In this paper, $q=0.01\;m^{2}/s^{3}$ which corresponds to an acceleration of $a\approx 1.8\;m/s^{2}$ for the considered system. The tracking filter is initialized when the person has reached the first reference position and is stationary. The filter is initialized using $m_{0}={\left[\begin{array}{cccc} x_{0}\;\left(m\right) & 0\;\left(m/s\right) & y_{0}\;\left(m\right) & 0\;\left(m/s\right) \end{array}\right]}^{T}$, where $x_{0}$ and $y_{0}$ are the center coordinates of the monitored area and $P_{0}=I_{4}$. To note, the ground truth position is never at ${\left[\begin{array}{cc} x_{0} & y_{0} \end{array}\right]}^{T}$ when the filter is initialized. Occupancy assessment is an important problem in DFLT [32] but for simplicity, we assume we know the time instances when the person enters and exits the monitored area.

The filters are evaluated using the root-mean-square error (RMSE) which is defined as $\mathrm{RMSE}=\sqrt{\mathrm{MSE}}$. The mean-squared error (MSE) is

(31) $$\mathrm{MSE}=\frac{1}{K}\sum_{k=1}^{K}{∥p_{k}-{\hat{p}}_{k}∥}_{2}^{2},$$

where K≈ 62,000 is the total number of estimates in one trial, $p_{k}$ denotes the ground truth position, the hat accent indicates the estimate and ${∥\cdot ∥}_{2}^{2}$ the square of the Euclidean norm.

## 6. Experimental Results

Matlab code to run the tracking and localization, smoothing and parameter estimation algorithms presented in Section 3 and Section 4, and the experimental data presented in Section 5 are available in [21]. The reader is referred to the associated readme file for an overall algorithmic description of the derivations presented in this paper and to the Matlab files for the actual implementation of the algorithms. The development efforts of this paper are experimentally validated in the following and benchmarked against existing solutions from literature. For now, $\mu_{0}$ is calculated using a two-minute empty-room calibration period. From Section 6.3 onward, $\mu_{0}$ is initialized without an empty-room calibration period.

### 6.1. EM with Existing DFLT Methods

In this section, it is shown that the EM algorithm can be used to enhance not only the performance of the proposed system, but also two de facto DFLT methods from literature. The first is RTI. The target is positioned as presented in Section 3.2, a standard Kalman filter (KF) is used for tracking and the RTSS is used for smoothing. The second method is a particle filter (PF). The implemented PF is a sequential importance resampling (SIR) filter with 1000 particles. The state estimate and covariance are calculated from the filtering distribution which is approximated by the set of particles and associated weights. A particle smoother could be implemented for approximating the smoothing distributions but for simplicity, the RTSS presented in Section 4.1 is used instead. A re-initialization procedure is required by the PF, because it is prone to diverge when the measurement model is inaccurate [3]. The PF is re-initialized, if the position error is larger than two meters, by drawing new particles from a uniform distribution within the monitored area and with zero velocity.

**Figure 6:**
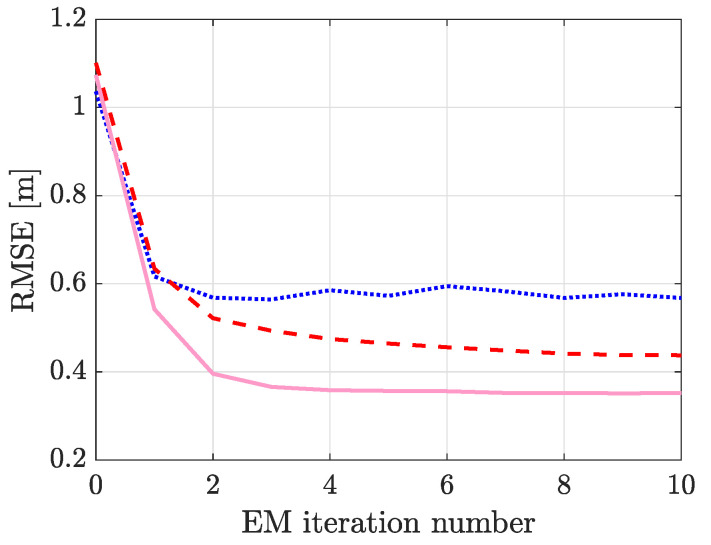
The RMSE, averaged over the 18 different experiments, as a function of EM iteration number. The DFLT methods are: EKF (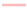), RTI (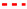) and PF (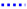).

In Figure 6, RMSE of the different filters as a function of EM iteration number. As shown, the tracking performance is satisfactory with the initial parameter estimates (EM iteration 0) and all filters have an RMSE above one meter. It is to be noted that most DFLT systems are implemented similarly, i.e., an empty-room calibration period is used to estimate $\mu_{0}$ and an educated guess is used for the other parameters. The empty-room data cannot be used to estimate the other parameters since they depend on the location of the person. However, they can be estimated using the state estimates after the person has entered the monitored area and moves around. In this paper, the EM algorithm based on Gaussian smoothing is used and with better parameter estimates, the tracking accuracy can be improved as we demonstrate in the following.

After the filtering recursion, the RTSS recursion starts from the last time step and proceeds backwards to the first time step. Thereafter, the E-step of the EM algorithm can be approximated using the smoothing distribution and the parameter estimates are obtained from the M-step in closed form. Using the new parameter estimates, the filtering recursion is started from the beginning. This iterative process improves the model parameter estimates and results in enhanced tracking accuracy. As shown in Figure 6, the RMSE decreases by 46–67%, depending on the filter. The results demonstrate that the implemented smoother and EM algorithm can also be used with other DFLT methods, and it is an effective method to improve system performance.

### 6.2. Parameter Estimation Algorithms

In this section, the EM algorithm is compared to the nonlinear least-squares (NLS) approach proposed in [22]. The parameter estimates are obtained by minimizing the cost function

(32) $$J\left(\Theta \right)=\sum_{k=1}^{K}{\left(y_{l,k}-h_{l}\left(m_{k}^{s},\Theta \right)\right)}^{2},$$

where $h_{l}\left(m_{k}^{s},\Theta \right)=\mu_{l}+\phi_{l}\exp\left(\Delta_{l,k}/\lambda_{l}\right)$ is the nonlinear exponential model and $\lambda_{l}$ is now a parameter to be estimated. In this paper, a nonlinear least-squares solver based on the interior-reflective Newton method described in [48] is used to find the minimum of J(Θ) and thereafter, the ML estimate of $\sigma_{l}^{2}$ is computed. The NLS approach provides freedom in the set of parameters that are estimated. In the following, we evaluate the NLS approach that estimates the following parameters $\Theta^{\left\{j\right\}}$: (i) $\Theta^{\left\{1\right\}}=\left[\mu ,\phi ,\sigma^{2}\right]$, as proposed in this paper; (ii) $\Theta^{\left\{2\right\}}=\left[\mu ,\phi ,\lambda \right]$, as proposed in [22]; and (iii) $\Theta^{\left\{3\right\}}=\left[\mu ,\phi ,\lambda ,\sigma^{2}\right]$, a system that estimates all measurement model parameters. The results are compared to the EM algorithm that estimates $\Theta^{\left\{1\right\}}$. We denote the parameter estimation algorithm and set of parameters simply as NLS($\Theta^{\left\{j\right\}}$).

**Figure 7:**
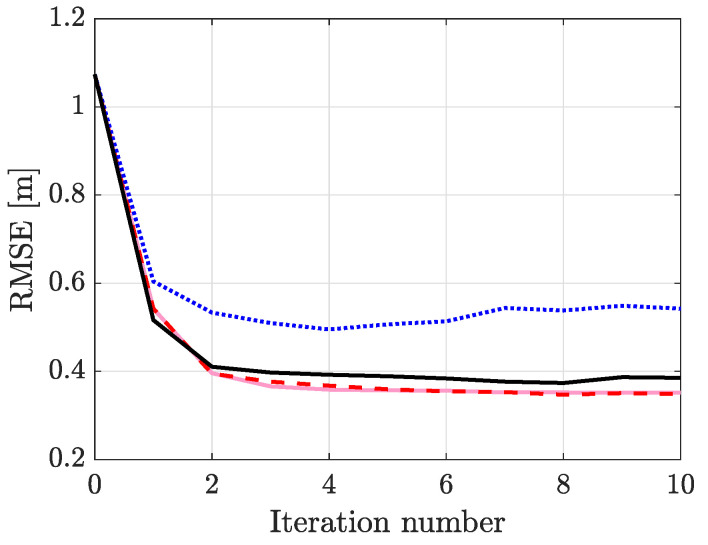
Comparison of different parameter estimation algorithms as a function of iteration number: EM($\Theta^{\left\{1\right\}}$) (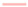), NLS($\Theta^{\left\{1\right\}}$) (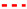), NLS($\Theta^{\left\{2\right\}}$) (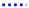) and NLS($\Theta^{\left\{3\right\}}$) (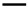).

The RMSEs are illustrated in Figure 7 and the results imply that estimating $\Theta^{\left\{1\right\}}$ yields the highest tracking accuracy whereas estimating $\Theta^{\left\{2\right\}}$ the lowest. To examine this difference more closely, we concentrate on Ex3 and the NLS and calculate the average $R^{2}$ statistic, defined as

(33) $$R^{2}=\sum_{l=1}^{L}\left(1-\frac{\sum_{k=1}^{K}{\left(y_{l,k}-h_{l}\left(m_{k}^{s},\Theta \right)\right)}^{2}}{\sum_{k=1}^{K}{\left(y_{l,k}-{\bar{y}}_{l}\right)}^{2}}\right)\cdot 100\%$$

and ${\bar{y}}_{l}=\frac{1}{K}\sum_{k=1}^{K}y_{l,k}$. The $R^{2}$ statistic measures how much of the observed variation in the mean can be explained by the model. For the two cases, $R^{2}=17.0\%$ and $R^{2}=19.1\%$ for NLS($\Theta^{\left\{1\right\}}$) and NLS($\Theta^{\left\{2\right\}}$) in corresponding order, meaning that estimating $\Theta^{\left\{2\right\}}$ explains the mean of the data more accurately, but the difference is only 2.1%. Calculating the Kullback–Leibler Divergence (KLD) yields 0.04 and 0.52 for $\Theta^{\left\{1\right\}}$ and $\Theta^{\left\{2\right\}}$ in respective order. As the KLD indicates, $\Theta^{\left\{2\right\}}$ is unable to account for the noise in the data and improved estimators can be developed when better noise models are available. Thus, estimating $\sigma^{2}$ rather than λ has a significantly higher impact on tracking accuracy. It is to be noted that EM($\Theta^{\left\{1\right\}}$) and NLS($\Theta^{\left\{1\right\}}$) yield comparative performance and small differences are expected, for example, due to the termination rule of the optimization method. Interestingly, NLS($\Theta^{\left\{3\right\}}$) has a higher RMSE than NLS($\Theta^{\left\{1\right\}}$). This is either caused by over fitting the model or then the optimization algorithm converges to a local minimum. In the measurement model, ϕ and λ are coupled and the optimization algorithm must solve for these simultaneously which can be problematic.

The main benefit of the proposed EM algorithm is that it can be solved in closed form using simple arithmetic operations, whereas the NLS approach requires a solver for the nonlinear optimization problem. In practice, the estimates only require computing two vector products (B and D) with complexity $O(K^{2})$, and calculating G with complexity $O(K^{2}+n^{3}K)$, where *n* is the state dimension and *K* the number of measurements. As an example, for three minutes of experimental data and using the initial parameter estimates, the computation time of the parameter estimation algorithms in experiment Ex3 are: [1.66,15.56,29.15,30.99]seconds for EM($\Theta^{\left\{1\right\}}$), NLS($\Theta^{\left\{1\right\}}$), NLS($\Theta^{\left\{2\right\}}$) and NLS($\Theta^{\left\{3\right\}}$) in respective order. The results are obtained using a Matlab implementation and a computer equipped with a 2.60 GHz Intel Core i7-8850H processor and 32 GB of RAM. As demonstrated by the results, the EM algorithm is computationally very efficient, up to 18 times faster than NLS. It is to be noted that the computation time of NLS has a significant dependence on the parameter values that are used to initialize the optimization algorithm. For example, the computation time of NLS($\Theta^{\left\{3\right\}}$) is 13.80s during the last parameter estimation iteration. Additionally, the link number has an impact since it defines the number of times NLS is called. As an example, the computation time in Ex1.1. and NLS($\Theta^{\left\{3\right\}}$) is 3.12s, which is significantly shorter than in Ex3.1. because the experiment only uses one frequency channel.

### 6.3. System Comparison

In this section, the proposed system is benchmarked against an adaptive radio tomographic imaging (ARTI) system [22]. ARTI is an imaging method that estimates μ and ϕ online, smoothing is used to enhance the image and state estimates, and NLS is used for estimating ϕ and λ. In the experiments, both systems are initialized without any prior information of the RSS, model parameters or location of the person. ARTI has an online calibration unit to estimate the reference RSS and the system is functional from the very beginning. The proposed system does not have such a feature and we use the online calibration unit of ARTI to estimate μ during the first filtering recursion and then the unit is disabled during the subsequent iterations. It is to be noted that μ could be initialized in various ways, but for matter of fairness we use the same method as ARTI uses.

The results are summarized in Table 2 and for each experiment, the results are averaged over the three trials. As shown, the proposed algorithm results in superior performance, an average decrease of 42% in the RMSE with respect to ARTI. For ARTI the estimates are most of the time accurate, but in certain positions, the location estimate is widely off (the skewness is 5 and kurtosis is 37 indicating that the distribution has a positive skew, and it is heavy tailed). The main reason for the large position errors is that a link can measure a really large RSS change when the person is not on the link line, the straight imaginary line between the TX and RX. When using imaging methods, this one link will dominate over the other links and the person will be localized in between the wrong TX-RX pair. The proposed system is not as vulnerable to such outliers (skewness is 2 and kurtosis is 10) because of the implemented measurement selection logic which discards RTI position estimates with abnormally large errors. The performance difference between ARTI and EKF can also be explained with the set of parameters that are estimated by the systems. ARTI estimates $\Theta^{\left\{2\right\}}=\left[\mu ,\phi ,\lambda \right]$ and this limits the achievable accuracy of the system as discussed in the previous section. To support this claim, in Figure 6 it is shown that an RTI solution together with EM almost achieves the same accuracy as the proposed solution.

### 6.4. System Performance over Time

**Figure 8:**
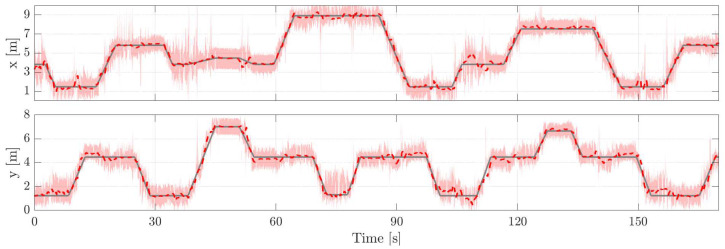
Tracking accuracy of the system in Ex6.3. In the figure, the ground truth coordinates are shown using (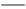), the estimated with (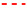) and the pink area illustrates the 3σ confidence interval of RTI position estimates.

Next, we demonstrate that the proposed system can maintain its high accuracy over time. The conducted trials are actually snippets from a longer experiment. The entire experiment contains the three trials explained before and a five-minute period when the person randomly walks inside the monitored area. In between the occupancy time periods, the person leaves the area for two minutes at a time. The five-minute period takes place before the trials, and we will run ten iterations of the EM algorithm using data from this period. Then, the obtained parameter estimates are used the next time the person enters the area. After each trial, the EM algorithm is used once to recalculate the model parameter estimates. The results are summarized in Table 3 and in every experiment, the tracking accuracy remains high throughout the different trials and there is no indication that the RMSE increases. This implies that the proposed system is suitable for estimating the model parameters without requiring human intervention and for maintaining high tracking accuracy over time. The ground truth trajectory together with the coordinate estimates is illustrated in Figure 8 for Ex6.3. Please note that the covariance of RTI position estimates changes from frame to frame and, therefore, the pink area which illustrates the 3σ confidence interval is not constant.

The described procedure is one of the possibilities how the proposed system would be used in practice, i.e., the parameters would be estimated at regular intervals or once the person has covered enough distance. However, there is a downside to the EM algorithm. It does not account for prior information, and it computes the ML estimates of the parameters from the data that is used as input. As an example, the data from the five-minute period is forgotten when the parameters are re-estimated after the first trial. This is an issue that must be solved for systems that are deployed over an extended period of time. One alternative is to compute the maximum a posteriori (MAP) estimates which can be done in practice by maximizing $Q\left(\Theta ,\Theta^{\left(i\right)}\right)+\log p\left(\Theta \right)$ at the M-step instead of the plain $Q(\Theta ,\Theta^{\left(i\right)})$ [36]. The prior information is included in the MAP estimate via the additional term logp(Θ).

The ground truth trajectory is reconstructed from the video recording. When the person is stationary, the ground truth locations are accurate because the reference positions were measured precisely with a laser rangefinder, and it is easy to extract these time instances from the video. When the person is moving, the ground truth can contain small errors because the video and measurements cannot be perfectly synchronized. Furthermore, the person does not move exactly with constant velocity. Let us assume that the ground truth trajectory is reconstructed accurately and let the null hypothesis be that the RMSE is the same when the person is stationary and moving. Then, we can test the statistical significance of the result in determine whether the null hypothesis should be rejected or retained. The RMSE is 32.8cm when the person is standing still and 33.0cm when moving. The *t*-test statistic of the independent two-sample *t*-test equals 0.0465 and the critical value is 2.03 with a 5% significance level. Since the statistic is lower than the critical value, the null hypothesis remains valid and the RMSE for stationary and moving periods can be considered the same. The result indicates that the ground truth trajectory has been accurately reconstructed.

### 6.5. Simulations

Lastly, we want to validate the development efforts of this paper numerically using simulations. Thus, the performance of the proposed system and ARTI are numerically analyzed using a simulation scenario which replicates Experiment 3. In total, 100 Monte Carlo simulations are performed and for each run, the model parameters are randomly drawn. The model parameters used in the simulation are drawn from: a Gaussian distribution, $\mu ∼N\left(-62.80,7.50\right)$, non-standardized Student’s t-distribution $\phi ∼T\left(-2.14,3.60,4.59\right)$, a uniform distribution $\lambda ∼U\left(0.01,0.13\right)$ and a log-normal distribution $\sigma^{2}∼L\left(0.79,0.88\right)$. The exact distributions of the model parameters are unknown, but the used ones provide a functional fit and they resemble the empirical distributions obtained using data from the open environment experiments. In the following, the RMSE is evaluated with respect to the posterior Cramér-Rao bound (PCRB) of RSS-based DFLT [3]. In addition, the RMSE of the parameter estimates are examined.

**Figure 9:**
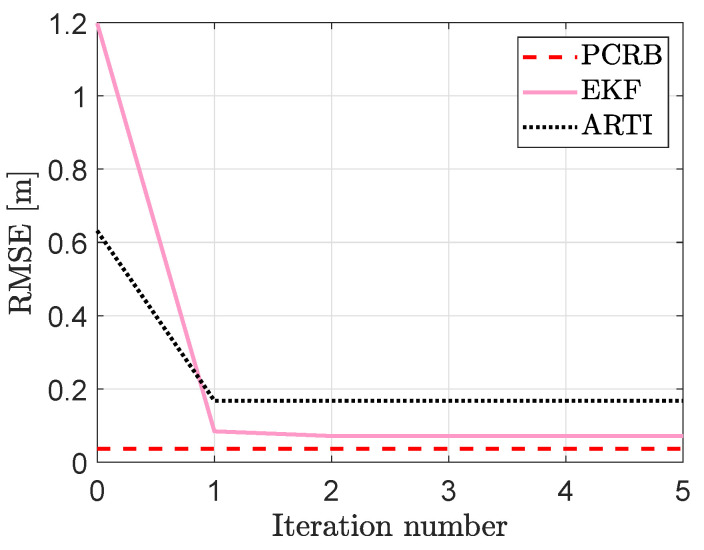
The posterior Cramér-Rao bound (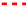) and RMSE of the proposed system (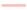) and the benchmark system (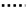). For every iteration number, the results are averaged over 100 Monte Carlo simulations.

In Figure 9, RMSE of the two systems as a function of parameter estimation iteration number is illustrated. With the initial parameter estimates, see iteration number zero in Figure 9, ARTI achieves a lower RMSE because μ and ϕ are estimated online during the filtering recursion. However, the proposed system outperforms ARTI after the parameters have been estimated by the EM and NLS algorithms. As illustrated in the figure, the EKF converges much closer to the PCRB than ARTI. More quantitatively, at iteration number five, the PCRB is 3.7cm whereas the RMSE of the EKF and ARTI are 7.1cm and 16.7cm in respective order, a 57% decrease in tracking error in favor of the EKF. The EKF achieves higher tracking accuracy due to two reasons. First, the EKF-based tracking algorithm is more accurate than the KF-based tracking algorithm of ARTI. With improved tracking performance the parameter estimates are more accurate which improves the tracking performance even further. As shown in Table 4, the RMSE of the parameter estimates for the EKF are significantly lower for μ, ϕ and $\sigma^{2}$. The second reason is that an accurate estimate of $\sigma^{2}$ rather than λ has a significantly higher impact on tracking accuracy as discussed in Section 6.2. As tabulated in Table 4, the RMSE of λ for ARTI and the EKF are 0.0365m and 0.0433m in respective order, only a 19% increase in RMSE when it is not estimated by the proposed system. Respectively, the RMSE of $\sigma^{2}$ decreases by 85% when estimated by the EM algorithm and as a result, improved estimators can be developed when better noise models are available.

## 7. Conclusions

The work in this paper addresses three fundamental challenges in DFLT: first, having an accurate model of the RSS as a function of target and transceiver positions; second, estimating the parameters of the model without requiring calibration or supervised training; and third, maintaining that model over time without requiring recalibration of the system. These problems are tackled by developing a system for estimating the RSS model parameters and the target’s arbitrary trajectory. In the paper, the model parameters are estimated using an EM algorithm based on Gaussian smoothing and a novel localization-and-tracking system is presented to fully use the EM algorithm’s potential. The system is validated using 18 different measurement data sets from two different environments. The results suggest that high tracking accuracy can be achieved without using calibration data. With respect to another adaptive DFLT system, it is demonstrated that the proposed system reduces the RMSE by 42%, while the parameter estimation algorithm is up to 18 times faster to compute. The developments of this paper streamline the deployment and maintenance needs of DFLT systems without sacrificing accuracy.

## Figures and Tables

**Table 1:** Imaging parameters.

Parameter		Unit
Pixel Variance (9)	$\sigma_{b}^{2}$	0.0005 (dB${}^{2}$)
Correlation distance (9)	$\delta_{d}$	0.5 (m)
Spatial decay rate (10)	λ	0.04 (m)
Pixel width (7)	$\delta_{p}$	0.25 (m)
Image threshold (12)	γ	0.70

**Table 2:** DFLT system comparison: RMSE ± standard deviation of the estimation error in centimeters.

	EKF	ARTI
Ex1 (open)	31.3±18.7	62.8±46.8
Ex2 (open)	20.8±11.4	26.0±15.0
Ex3 (open)	17.2±8.9	23.8±11.5
Ex4 (apt.)	50.4±28.0	85.1±62.0
Ex5 (apt.)	40.1±20.9	62.9±41.3
Ex6 (apt.)	36.7±21.1	49.6±28.4

**Table 3:** RMSE in centimeters of the proposed system in the different experiments and trials.

	Trial 1	Trial 2	Trial 3	Average
Ex1 (open)	23.4	25.1	28.2	25.6
Ex2 (open)	21.7	16.8	19.2	19.2
Ex3 (open)	18.3	16.3	16.9	17.2
Ex4 (apt.)	57.9	54.9	49.3	54.0
Ex5 (apt.)	42.8	41.5	39.7	41.3
Ex6 (apt.)	39.2	42.7	39.0	40.3

**Table 4:** RMSE of parameter estimates.

	$\hat{\mu }$ [dB]	$\hat{\phi }$ [dB]	$\hat{\lambda }$ [m]	${\hat{\sigma }}^{2}$ [dB${}^{2}$]
EKF	0.1705	1.3688	0.0433	0.6324
ARTI	0.6623	2.5230	0.0365	4.1551

## Data Availability

The experiments conducted in this paper, together with Matlab code to run the presented filtering, smoothing and EM algorithms are made publicly available and are published in [21].

## Appendix A. EM Algorithm

The parameter updates (27) for the linear-in-parameters measurement model (26) are derived in the following. In state-space models, $Q(\Theta ,\Theta^{\left(i\right)})$ can be decomposed to [36,37,38]

(A1) $$\begin{array}{cc} Q(\Theta ,\Theta^{\left(i\right)}) & =\sum_{k=1}^{K}E\left\{\log p\left(y_{k}|x_{k},\Theta \right)|y_{1:K}\right\}\\ & +\sum_{k=1}^{K}E\left\{\log p\left(x_{k}|x_{k-1},\Theta \right)|y_{1:K}\right\}\\ & +E\left\{\log p\left(x_{0}|\Theta \right)|y_{1:K}\right\}, \end{array}$$

by making use of the Markov property of the state sequence and conditional independence of the measurements. Noting that in DFLT, the dynamic model $p(x_{k}|x_{k-1})$ and initial distribution $p(x_{0})$ are independent of the unknown parameters, maximizing (A1) is equivalent to maximizing the first term only, i.e., maximizing (25). To simplify the notation, the dependence of $h_{l,k}$ on $x_{k}$ is not explicitly stated in the following. The log-likelihood of the first term is

$$\begin{array}{cc} \; & \log p\left(y_{k}|x_{k},\Theta \right)=\sum_{l=1}^{L}\log N\left(y_{l,k}|h_{l,k}\theta_{l},\sigma_{l}^{2}\right)\\ \; & =\sum_{l=1}^{L}\left(-\frac{1}{2}\log\left(2\pi \right)-\frac{1}{2}\log\left(\sigma_{l}^{2}\right)-\frac{1}{2\sigma_{l}^{2}}{∥y_{l,k}-h_{l,k}\theta_{l}∥}^{2}\right), \end{array}$$

and taking the derivatives with respect to $\theta_{l}$ and $\sigma_{l}^{2}$ yields

$$\begin{array}{cc} \nabla_{\theta_{l}}\log p\left(y_{k}|x_{k},\Theta \right) & =\frac{1}{\sigma_{l}^{2}}h_{l,k}^{T}\left(y_{l,k}-h_{l,k}\theta_{l}\right),\\ \nabla_{\sigma_{l}^{2}}\log p\left(y_{k}|x_{k},\Theta \right) & =-\frac{1}{2\sigma_{l}^{2}}+\frac{1}{2\sigma_{l}^{4}}{∥y_{l,k}-h_{l,k}\theta_{l}∥}^{2}. \end{array}$$

To estimate the parameters, (25) is approximated using the smoothing distribution provided by the Gaussian smoother in Section 4.1 and given by

(A2) $$p\left(x_{k}|y_{1:K},\Theta^{\left(i\right)}\right)\approx N\left(x_{k};m_{k}^{s},P_{k}^{s}\right).$$

The maximization step is computed by setting the gradient with respect to $\theta_{l}$ to zero, i.e.,

(A3) $$\begin{array}{cc} \nabla_{\theta_{l}}Q\left(\Theta ,\Theta^{\left(i\right)}\right) & =\sum_{k=1}^{K}E\left\{\nabla_{\theta_{l}}\log p\left(y_{k}|x_{k},\Theta \right)|y_{1:K}\right\}\\ \approx \sum_{k=1}^{K}\frac{1}{\sigma_{l}^{2}} & E\left\{h_{l,k}^{T}y_{l,k}-h_{l,k}^{T}h_{l,k}\theta_{l}|y_{1:K}\right\}=0. \end{array}$$

Using the notation in (28) and solving (A3) for $\theta_{l}$ yields

(A4) $${\hat{\theta }}_{l}=G^{-1}B.$$

Similarly, for $\sigma_{l}^{2}$ we obtain

(A5) $$\begin{array}{cc} \; & \nabla_{\sigma_{l}^{2}}Q\left(\Theta ,\Theta^{\left(i\right)}\right)=\sum_{k=1}^{K}E\left\{\nabla_{\sigma_{l}^{2}}\log p\left(y_{k}|x_{k},\Theta \right)|y_{1:K}\right\}\\ \; & \approx \frac{-K}{2\sigma_{l}^{2}}+\frac{1}{2\sigma_{l}^{4}}(\sum_{k=1}^{K}y_{l,k}^{T}y_{l,k}-2\left[\sum_{k=1}^{K}y_{l,k}^{T}E\left\{h_{l,k}|y_{1:K}\right\}\right]\theta_{l}\\ \; & \;\;+\theta_{l}^{T}\left[\sum_{k=1}^{K}E\left\{h_{l,k}^{T}h_{l,k}|y_{1:K}\right\}\right]\theta_{l})=0. \end{array}$$

Using the notation in (28), substituting $\theta_{l}$ to (A5) and solving for $\sigma_{l}^{2}$ yields

(A6) $${\hat{\sigma }}_{l}^{2}=\frac{1}{K}\left(D-B^{T}G^{-1}B\right).$$

Finally, note that the optimal ${\hat{\theta }}_{l}$ does not depend on ${\hat{\sigma }}_{l}^{2}$ so that they can be sequentially optimized by first solving for ${\hat{\theta }}_{l}$ and then substituting the value into the estimate of ${\hat{\sigma }}_{l}^{2}$.

## References

[1] Monekosso D., Florez-Revuelta F., Remagnino P. (2015). Ambient Assisted Living [Guest editors’ introduction]. IEEE Intell. Syst..

[2] Wilson J., Patwari N. (2010). Radio Tomographic Imaging with Wireless Networks. IEEE Trans. Mob. Comput..

[3] Kaltiokallio O., Hostettler R., Patwari N. (2021). A Novel Bayesian Filter for RSS-based Device-free Localization and Tracking. IEEE Trans. Mob. Comput..

[4] Mager B., Patwari N., Bocca M. Fall detection using RF sensor networks. Proceedings of the IEEE 24th Annual International Symposium on Personal, Indoor, and Mobile Radio Communications.

[5] Adib F., Mao H., Kabelac Z., Katabi D., Miller R.C. (2015). Smart Homes That Monitor Breathing and Heart Rate. Proceedings of the 33rd Annual ACM Conference on Human Factors in Computing Systems.

[6] Pu Q., Gupta S., Gollakota S., Patel S. (2013). Whole-Home Gesture Recognition Using Wireless Signals. Proceedings of the 19th Annual International Conference on Mobile Computing & Networking.

[7] Hsu C.Y., Hristov R., Lee G.H., Zhao M., Katabi D. (2019). Enabling Identification and Behavioral Sensing in Homes Using Radio Reflections. Proceedings of the 2019 CHI Conference on Human Factors in Computing Systems.

[8] Wilson J., Patwari N. (2011). See-Through Walls: Motion Tracking Using Variance-Based Radio Tomography Networks. IEEE Trans. Mob. Comput..

[9] Hsu C.Y., Liu Y., Kabelac Z., Hristov R., Katabi D., Liu C. (2017). Extracting Gait Velocity and Stride Length from Surrounding Radio Signals. Proceedings of the 2017 CHI Conference on Human Factors in Computing Systems.

[10] Xu C., Firner B., Zhang Y., Howard R., Li J., Lin X. Improving RF-based device-free passive localization in cluttered indoor environments through probabilistic classification methods. Proceedings of the 2012 ACM/IEEE 11th International Conference on Information Processing in Sensor Networks.

[11] Xu C., Firner B., Moore R.S., Zhang Y., Trappe W., Howard R., Zhang F., An N. SCPL: Indoor device-free multi-subject counting and localization using radio signal strength. Proceedings of the 2013 ACM/IEEE International Conference on Information Processing in Sensor Networks.

[12] Mager B., Lundrigan P., Patwari N. (2015). Fingerprint-Based Device-Free Localization Performance in Changing Environments. IEEE J. Sel. Areas Commun..

[13] Savazzi S., Nicoli M., Carminati F., Riva M. (2014). A Bayesian Approach to Device-Free Localization: Modeling and Experimental Assessment. IEEE J. Sel. Top. Signal Process..

[14] Kaltiokallio O., Bocca M., Patwari N. Follow @grandma: Long-term device-free localization for residential monitoring. Proceedings of the 37th Annual IEEE Conference on Local Computer Networks-Workshops.

[15] Savazzi S., Rampa V., Vicentini F., Giussani M. (2016). Device-Free Human Sensing and Localization in Collaborative Human–Robot Workspaces: A Case Study. IEEE Sens. J..

[16] Yiğitler H., Jäntti R. Experimental accuracy assessment of radio tomographic imaging methods. Proceedings of the 2016 IEEE International Conference on Pervasive Computing and Communication Workshops.

[17] Nannuru S., Li Y., Zeng Y., Coates M., Yang B. (2013). Radio-Frequency Tomography for Passive Indoor Multitarget Tracking. IEEE Trans. Mob. Comput..

[18] Guo Y., Huang K., Jiang N., Guo X., Li Y., Wang G. (2015). An Exponential-Rayleigh Model for RSS-Based Device-Free Localization and Tracking. IEEE Trans. Mob. Comput..

[19] Kianoush S., Savazzi S., Vicentini F., Rampa V., Giussani M. (2017). Device-Free RF Human Body Fall Detection and Localization in Industrial Workplaces. IEEE Internet Things J..

[20] Li Y., Chen X., Coates M., Yang B. Sequential Monte Carlo Radio-Frequency tomographic tracking. Proceedings of the 2011 IEEE International Conference on Acoustics, Speech and Signal Processing.

[21] Kaltiokallio O. (2021). Device-Free Localization and Tracking Data and Software.

[22] Kaltiokallio O., Jäntti R., Patwari N. (2017). ARTI: An Adaptive Radio Tomographic Imaging System. IEEE Trans. Veh. Technol..

[23] Zhao Y., Patwari N. (2015). Robust Estimators for Variance-Based Device-Free Localization and Tracking. IEEE Trans. Mob. Comput..

[24] Edelstein A., Rabbat M. (2013). Background Subtraction for Online Calibration of Baseline RSS in RF Sensing Networks. IEEE Trans. Mob. Comput..

[25] Huang K., Guo Y., Guo X., Wang G. (2014). Heterogeneous Bayesian compressive sensing for sparse signal recovery. IET Signal Process..

[26] Huang K., Tan S., Luo Y., Guo X., Wang G. (2017). Enhanced radio tomographic imaging with heterogeneous Bayesian compressive sensing. Pervasive Mob. Comput..

[27] Zheng Y., Men A. Through-wall tracking with radio tomography networks using foreground detection. Proceedings of the 2012 IEEE Wireless Communications and Networking Conference.

[28] Men A., Xue J., Liu J., Xu T., Zheng Y. Applying background learning algorithms to radio tomographic imaging. Proceedings of the 2013 16th International Symposium on Wireless Personal Multimedia Communications.

[29] Zhao Y., Patwari N., Phillips J.M., Venkatasubramanian S. Radio tomographic imaging and tracking of stationary and moving people via kernel distance. Proceedings of the 2013 ACM/IEEE International Conference on Information Processing in Sensor Networks.

[30] Hillyard P., Luong A., Patwari N. Highly Reliable Signal Strength-Based Boundary Crossing Localization in Outdoor Time-Varying Environments. Proceedings of the 2016 15th ACM/IEEE International Conference on Information Processing in Sensor Networks.

[31] Al-Husseiny A., Patwari N. Unsupervised Learning of Signal Strength Models for Device-Free Localization. Proceedings of the 2019 IEEE 20th International Symposium on “A World of Wireless, Mobile and Multimedia Networks”.

[32] Hillyard P., Patwari N. (2020). Never Use Labels: Signal Strength-Based Bayesian Device-Free Localization in Changing Environments. IEEE Trans. Mob. Comput..

[33] Chen X., Edelstein A., Li Y., Coates M., Rabbat M., Men A. Sequential Monte Carlo for simultaneous passive device-free tracking and sensor localization using received signal strength measurements. Proceedings of the 10th ACM/IEEE International Conference on Information Processing in Sensor Networks.

[34] Doucet A., Johansen A.M., Crisan D., Rozovskii B. (2011). A Tutorial on Particle Filtering and Smoothing: Fifteen Years Later. Handbook of Nonlinear Filtering.

[35] Schön T.B., Wills A., Ninness B. (2011). System identification of nonlinear state-space models. Automatica.

[36] Särkkä S. (2013). Bayesian Filtering and Smoothing.

[37] Kokkala J., Solin A., Särkkä S. Expectation maximization based parameter estimation by sigma-point and particle smoothing. Proceedings of the 17th International Conference on Information Fusion.

[38] Kokkala J., Solin A., Särkkä S. (2016). Sigma-Point Filtering and Smoothing Based Parameter Estimation in Nonlinear Dynamic Systems. J. Adv. Inf. Fusion.

[39] Yiğitler H., Kaltiokallio O., Hostettler R., Abrar A.S., Jäntti R., Patwari N., Särkkä S. (2020). RSS Models for Respiration Rate Monitoring. IEEE Trans. Mob. Comput..

[40] Bar-Shalom Y., Li X.R. (2001). Estimation with Applications to Tracking and Navigation.

[41] Kaltiokallio O., Yiğitler H. Movement Detection Using A Reciprocal Received Signal Strength Model. Proceedings of the ICASSP 2021-2021 IEEE International Conference on Acoustics, Speech and Signal Processing (ICASSP).

[42] Patwari N., Agrawal P. Effects of Correlated Shadowing: Connectivity, Localization, and RF Tomography. Proceedings of the 2008 International Conference on Information Processing in Sensor Networks.

[43] Martin R.K., Folkerts A., Heinl T. (2014). Accuracy vs. Resolution in Radio Tomography. IEEE Trans. Signal Process..

[44] Yiğitler H., Jäntti R., Kaltiokallio O., Patwari N. (2018). Detector Based Radio Tomographic Imaging. IEEE Trans. Mob. Comput..

[45] CC2531F128 (2010). A USB-Enabled System-on-Chip Solution for 2.4 GHz IEEE 802.15.4 and ZigBee Applications.

[46] (2003). IEEE 802.15.4-2003 Standard. http://www.ieee802.org/15/pub/TG4Expert.html.

[47] Bocca M., Kaltiokallio O., Patwari N., Chessa S., Knauth S. (2013). Radio Tomographic Imaging for Ambient Assisted Living. Evaluating AAL Systems through Competitive Benchmarking.

[48] Coleman T.F., Li Y. (1996). An Interior Trust Region Approach for Nonlinear Minimization Subject to Bounds. SIAM J. Optim..

